# Enhancing diffusion-weighted prostate MRI through self-supervised denoising and evaluation

**DOI:** 10.1038/s41598-024-75007-x

**Published:** 2024-10-16

**Authors:** Laura Pfaff, Omar Darwish, Fabian Wagner, Mareike Thies, Nastassia Vysotskaya, Julian Hossbach, Elisabeth Weiland, Thomas Benkert, Cornelius Eichner, Dominik Nickel, Tobias Wuerfl, Andreas Maier

**Affiliations:** 1 Pattern Recognition Lab, Friedrich-Alexander-Universität Erlangen-Nürnberg, 91058 Erlangen, Germany; 2 Magnetic Resonance, Siemens Healthineers AG, 91052 Erlangen, Germany

**Keywords:** Magnetic resonance imaging, Computer science, Biomedical engineering

## Abstract

Diffusion-weighted imaging (DWI) is a magnetic resonance imaging (MRI) technique that provides information about the Brownian motion of water molecules within biological tissues. DWI plays a crucial role in stroke imaging and oncology, but its diagnostic value can be compromised by the inherently low signal-to-noise ratio (SNR). Conventional supervised deep learning-based denoising techniques encounter challenges in this domain as they necessitate noise-free target images for training. This work presents a novel approach for denoising and evaluating DWI scans in a self-supervised manner, eliminating the need for ground-truth data. By leveraging an adapted version of Stein’s unbiased risk estimator (SURE) and exploiting a phase-corrected combination of repeated acquisitions, we outperform both state-of-the-art self-supervised denoising methods and conventional non-learning-based approaches. Additionally, we demonstrate the applicability of our proposed approach in accelerating DWI scans by acquiring fewer image repetitions. To evaluate denoising performance, we introduce a self-supervised methodology that relies on analyzing the characteristics of the residual signal removed by the denoising approaches.

## Introduction

Diffusion-weighted imaging (DWI) is a form of magnetic resonance imaging (MRI) that uses the rate of water diffusion within tissues to generate image contrast. DWI holds significant clinical relevance, particularly in stroke detection and tumor characterization, as it provides valuable insights into tissue microstructure^1,2^. To account for anisotropy, the water diffusion rate is commonly assessed in various directions. Instead of viewing these images separately, they are combined into a single trace image for diagnosis by calculating the geometric mean across all acquired directions^3^. The degree of diffusion weighting depends on the b-value which is determined by the strength and timing of the diffusion-sensitizing gradients^4^. DWI images are typically acquired using at least two b-values to enable the calculation of quantitative apparent diffusion coefficient (ADC) maps^5^.

Despite its diagnostic value, DWI faces a notable limitation arising from its inherently low signal-to-noise-ratio (SNR), particularly in images acquired with higher b-values. The SNR degradation is attributed to the diffusion weighting and extended echo times required to accommodate the additional gradient pulses^6^. Consequently, in smaller anatomical regions such as the prostate, individual DWI acquisitions offer limited diagnostic usability. This problem of low SNR is further exacerbated at low field strengths, which are gaining increasing attention in clinical MRI due to benefits such as improved patient comfort, reduced susceptibility artifacts, and lower operational costs^7^. However, the inherently lower SNR at these field strengths presents additional challenges in obtaining high-quality DWI images.

Addressing the challenge of SNR improvement in DWI, a common approach involves acquiring multiple repetitions and averaging them. This averaging can be performed either on the complex-valued reconstructed images before or after computing a magnitude image. When averaging after magnitude computation, the resulting magnitude images exhibit a higher noise floor caused by the Rician noise bias, as the noise distribution is not zero-centered^8^. Conversely, averaging before magnitude computation proves beneficial, as the noise in complex-valued reconstructed images follows a zero-centered Gaussian distribution^9^. However, there is a risk of signal cancellation due to phase instabilities across repetitions. One solution to mitigate this effect is to perform a phase correction on the complex-valued images prior to averaging^10,11^. Despite its potential benefits, the acquisition of a considerable number of repetitions is time-consuming, and constraints such as patient motion can impact the achievable SNR improvement through repetition averaging.

In addition to simple averaging, various conventional denoising techniques can be employed to further enhance the SNR and potentially reduce the number of required image repetitions. Non-local means (NLM) denoising is a widely used method for denoising medical images, including DWI^12,13^, that exploits the redundancy in image patches or repeated acquisitions to estimate the underlying noise-free signal. One popular approach that applies this principle is block-matching and 3D filtering (BM3D)^14^. It first groups similar patches in a 2D image and then performs collaborative filtering in a 3D transform domain to suppress noise while preserving important image structures. Techniques based on principal component analysis (PCA) further demonstrated practical utility for DWI denoising^15–17^. For instance, Veraart et al.^18^ introduced a PCA-based denoising method called Marchenko-Paskur principal component analysis (MPPCA) for DWI data, leveraging random matrix theory to selectively eliminate noise-only principal components.

With the advancements in deep learning, several approaches have been proposed to denoise DWI scans using convolutional neural networks (CNNs)^5,19–21^. Deep learning-based denoising strategies can typically be subdivided into supervised and self-supervised learning approaches. Supervised learning for denoising relies on noise-free training data to learn the mapping between noisy and noise-free data. For instance, Jurek et al.^19^ utilized simulated DWI data to generate a noise-free reference, enabling supervised training of their model. Kaye et al.^5^ proposed to accelerate prostate DWI acquisitions by using non-accelerated images obtained from the full repetition count as reference. They further introduce a guided approach that uses low b-value data as additional input to a CNN that is trained to denoise high b-value data.

In contrast, self-supervised learning approaches exploit the inherent properties or structure of the data to estimate and remove noise, making them well-suited for medical imaging scenarios where noise-free data is scarce. For instance, Noise2Noise^22^ is a self-supervised denoising technique that learns to remove noise from an image by training a neural network on pairs of noisy images without corresponding clean images, exploiting the property that the noise in different acquisitions of the same scene is uncorrelated. Wagner et al. introduced Noise2Contrast, a self-supervised method that utilizes data from multiple measured MRI contrasts to train a denoising model^23^.

Alternative approaches explore the incorporation of supplementary information to achieve results that closely resemble those attained through supervised training. Previous research efforts have suggested the utilization of Stein’s unbiased risk estimator (SURE)^24^ as a loss function for estimating the mean squared error (MSE) between the output image and an unknown ground truth. This approach incorporates the variance of the Gaussian noise distribution and has found application in diverse computer vision tasks^25–28^. Given the Gaussian distribution of noise in complex-valued reconstructed MRI^9^, and thus also DWI^10^, SURE emerges as a well-suited method for DWI denoising.

In our previous study^29^, we introduced an enhanced SURE-based methodology specifically designed for MRI applications, addressing spatially-variant noise amplification during parallel image reconstruction. This method utilized quantitative noise maps obtained from a dedicated noise adjustment scan, showing competitive performance compared to state-of-the-art supervised techniques using MSE loss and substantially outperforming conventional self-supervised approaches.

In this work, we build upon this foundation and make the following significant contributions:

1. We present a novel, fully self-supervised method for denoising prostate DWI that relies solely on the input images, eliminating the need for additional data such as noise prescans or scanner-specific information.
2. We demonstrate how incorporating noise prescans and supplementary scanner information can enhance the applicability of our proposed approach, providing flexibility in different scenarios.
3. We illustrate the impact of leveraging complementary information from multiple b-values to improve denoising performance,
4. We conduct comprehensive experiments on clinical DWI scans, showcasing the superior performance of our method compared to both state-of-the-art learning-based and non-learning-based techniques.
5. We introduce a new methodology for evaluating the performance of denoising methods in the absence of noise-free ground-truth data, addressing a significant challenge in self-supervised learning.

## Methods

### Data

An overview of the three different data sets used in this work is provided in Table 1.

**Table 1:** Overview of the three different data sets used in this work.

Source	Scans	Field strength	b-values	Repetitions	Directions	Split (train/val/test)
fastMRI	312	3 T	b=50 s/mm^2^ b=1000 s/mm^2^	4 12	3	218/48/46
Siemens Healthineers	255	1.5 T, 3 T	b=50 s/mm^2^ b=800 s/mm^2^	4 10	4	191/64/-
Siemens Healthineers	5	0.55 T	b=50 s/mm^2^ b=800 s/mm^2^	4 22	4	-/-/5

#### fastMRI data

 The first data set was obtained from the NYU fastMRI initiative database^30–32^. The fastMRI data set is an open-source data set which contains raw and DICOM data from MRI acquisitions of knees, brains, and prostate DWI. The prostate data set comprises 312 patient scans, with an average patient age of 66 ± 8 years. The clinical prostate scans were conducted using two 3 T scanners (MAGNETOM Vida, Siemens Healthineers, Erlangen, Germany) and were collected between March 2020 and April 2021 at NYU Langone Health (NYULH). All data underwent thorough anonymization to ensure privacy and compliance. Furthermore, the curation of this data set was carried out as part of a study that received approval from the NYU School of Medicine Institutional Review Board. The NYU fastMRI investigators supplied the data but were not involved in the analysis or the writing of this work.

**Fig. 1:**
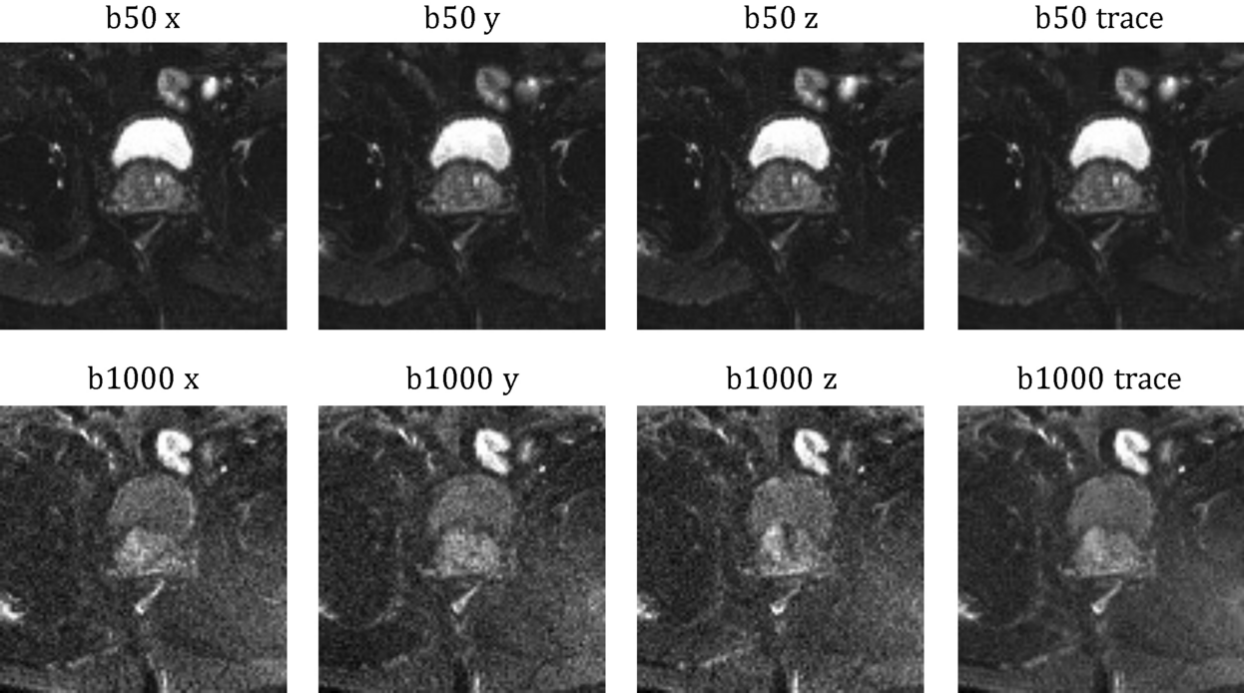
Representative averaged diffusion-weighted prostate images from the fastMRI data set for both b-values b50 and b1000 in the x, y, and z-directions are presented along with their corresponding trace images.

The 312 patient scans were divided into training/validation/test sets with 218/48/46 patients, respectively. For each patient, T2-weighted turbo spin echo (TSE) and echo planar imaging (EPI) DWI sequences were acquired. During the EPI-DWI scans, b-values of 50 s/mm^2^ (b50) and 1000 s/mm^2^ (b1000) were obtained along three diffusion encoding directions, involving four and twelve repetitions, respectively. The averaged repetitions of each direction as well as the respective trace-weighted images (geometric mean) for b50 and b1000 are presented in Fig. 1. DWI images acquired with higher b-values inherently contain less signal due to increased sensitivity to diffusion effects^6^. The higher b-values lead to more pronounced attenuation of the signal, resulting in reduced SNR. Thus, despite being averaged over fewer repetitions, the presented b50 images exhibit enhanced SNR and improved delineation of anatomical structures compared to the b1000 images.

For our experiments, the raw data was reconstructed using a slightly adapted version of the code repository made publicly available by the fastMRI research team^32^. Rather than employing the complete reconstruction pipeline, we retrieved the individual repetitions in complex-valued reconstructed format.

#### In-house data

 We further employed two more data sets to showcase 1) the improved applicability of our method having access to supplementary information from the scanner, and 2) the robustness of our proposed method when applied to unseen image features. The first data set comprised 255 prostate DWI scans from volunteers acquired with 1.5 T and 3 T scanners (MAGNETOM, Siemens Healthineers, Erlangen, Germany) using standardized protocols. The data set was split into 191 scans for training and 64 scans for validation. Each scan involved two b-values, b=50 s/mm^2^ (b50) and b=800 s/mm^2^ (b800), with four diffusion directions each. Four repetitions were collected for b50, and ten repetitions for b800.

The corresponding test set consisted of five DWI prostate scans acquired from volunteers using a 0.55 T scanner (MAGNETOM Free.Max, Siemens Healthineers, Erlangen, Germany). In standard low-field (B0 < 1.0 T) DWI scans, we typically perform four repetitions for b50 and 22 repetitions for b800.

The in-house MRI measurements were conducted in strict accordance with all relevant ethical regulations, including the principles embodied in the declaration of Helsinki, as well as EU and German law. The responsible licensing authorities, i.e., the Berufsgenossenschaft Energie Textil Elektro Medienerzeugnisse (BG ETEM, IK 120590446) Cologne, Germany, as well as the Gewerbeaufsichtsamt (Regierung von Mittelfranken) approved the volunteer scanning for research purposes under the registration number QR24-03_MR. All participants received an informed consent discussion and gave their written informed consent for their data being further used and processed.

### SURE for spatially variant noise

The purpose of SURE^24^ is to estimate the MSE between the unknown mean ${\textbf {x}} \in {\mathbb {R}}^{D}$ of a Gaussian-distributed signal ${\textbf {y}} \in {\mathbb {R}}^{D}$ and its prediction $\hat{{\textbf {x}}}=f({\textbf {y}})$. Previous studies^25,26^ have employed SURE for image denoising, wherein the unknown noise-free image ${\textbf {x}}$ is treated as the mean vector of the noisy image ${\textbf {y}}$ corrupted by additive, zero-mean Gaussian noise, denoted as ${\textbf {y}} \sim \ \mathscr {N}({\textbf {x}},\sigma ^2 {\textbf {I}})$, with ${\textbf {I}}$ representing the identity matrix.

The noise present in complex-valued reconstructed magnetic resonance (MR) images can be characterized as a spatially variant Gaussian distribution^9^. The spatial dependence of this noise is a result of the anisotropic amplification of noise during the image reconstruction process, particularly in the context of parallel imaging. In order to effectively account for this spatially varying noise amplification, we introduced an adapted SURE methodology in previous work^29^. This adapted approach incorporates a noise map $\varvec{\sigma } \in {\mathbb {R}}^{D}$, which indicates the standard deviation of the noise for each pixel, as opposed to using a scalar noise level for the whole image.

The observed measurement vector ${\textbf {y}}$ is subject to a multivariate Gaussian distribution with the clean image ${\textbf {x}}$ as its mean and a covariance matrix $\varvec{\Sigma }$ that represents the additive noise $\varvec{\eta }\sim \ \mathscr {N}(0,\varvec{\Sigma })$. Here, $\varvec{\Sigma }$ can be understood as a diagonal matrix, where the individual entries are given by $\Sigma _{dd} = \sigma _d^2$.

Subsequently, the expectation of the MSE in the presence of spatially variant noise can be formulated using the SURE methodology:

1 $$\begin{aligned} \text {E}_{{\textbf {x}}}\Big \{\ \hspace{-0.3em} \frac{1}{D} \Vert f({\textbf {y}})-{\textbf {x}} \Vert ^2 \Big \}\ = \text {E}_{{\textbf {x}}}\Big \{\ \hspace{-0.3em} \frac{1}{D} \Bigl ( \Vert f({\textbf {y}})-{\textbf {y}} \Vert ^2 - \sum \nolimits _{d=1}^D\sigma _d^2 + 2\text {div}_{{\textbf {y}}} ( \varvec{\sigma ^2} \odot f({\textbf {y}})) \Bigr ) \Big \}\ , \end{aligned}$$

with $\odot$ denoting element-wise multiplication. In the context of denoising, we can proceed to train a neural network denoted as $f({\textbf {y}})$, which takes noisy measurements ${\textbf {y}}$ as input and predicts an estimation of ${\textbf {x}}$ as output. To estimate the expected value of the mean squared error (MSE), we leverage the SURE methodology. In practical terms, the expectation expressed in Equation 1 can be approximated by computing the loss across multiple images within a batch and subsequently averaging the results.

The initial expression $\Vert f({\textbf {y}})-{\textbf {y}} \Vert ^2$ is referred to as the *fidelity term*, which serves to minimize discrepancies between the observed input and the denoised predictions. In contrast, the *divergence term* $\text {div}_{{\textbf {y}}} ( \varvec{\sigma ^2} \odot f({\textbf {y}}))$ penalizes the model for deviating from its predictions when encountering minor alterations in the observations^28^. The third component, $\sum \nolimits _{d=1}^D\sigma _d^2$, is an additive constant which can be neglected for optimization.

Calculating the *divergence term* analytically can be notably inefficient, particularly when dealing with neural networks. Recognizing this challenge, Ramani et al. introduced MC-SURE, a Monte Carlo (MC) method designed to offer an estimation of the *divergence term*. Hence, we tailored the Monte Carlo (MC) estimator to suit our newly introduced spatially variant Gaussian SURE formulation:

2 $$\begin{aligned} \text {div}_{{\textbf {y}}} ( \varvec{\sigma ^2} \odot f({\textbf {y}})) \approx {\textbf {b}}^T \bigg (\ \hspace{-0.4em} \varvec{\sigma ^2} \hspace{-0.1em}\odot \hspace{-0.1em}\Big (\ \frac{f({\textbf {y}}+\varepsilon {\textbf {b}})-f({\textbf {y}})}{\varepsilon } \Big )\ \hspace{-0.5em} \bigg )\ . \end{aligned}$$

Here, ${\textbf {b}}$ represents a zero-mean, independently and identically distributed (i.i.d.) random vector with unit variance, while $\varepsilon$ represents a constant small value. As an example, $\varepsilon$ can be defined as $\varepsilon = \text {max}({\textbf {y}}) \cdot 10^{-3}$, as proposed by Metzler et al.^26^.

### Proposed pipelines

#### Using noise maps derived from the images

**Fig. 2:**
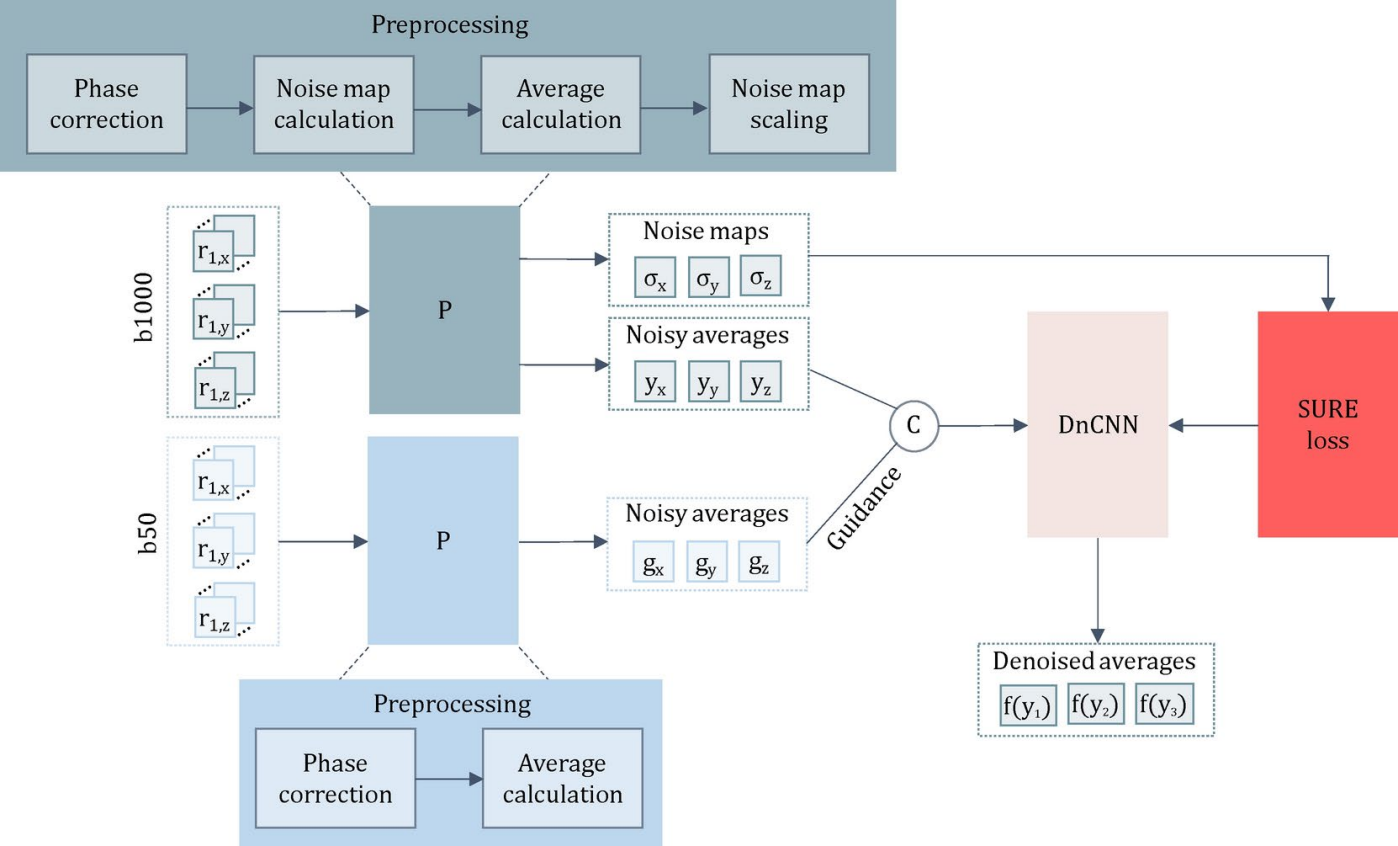
The proposed denoising pipeline using only image data. We start from the tri-directional image repetitions for two distinct b-values, b50 and b1000. During the preprocessing phase, we generate an averaged image for each direction x,y, and z by employing complex averaging alongside phase correction for both b-values. Corresponding noise maps are further computed for the b1000 images. The averaged images for b50 and b1000 are then concatenated along the channel dimension into a joint input for the denoising network DnCNN, where the b50 images serve as guidance. The noise maps are employed in the calculation of the SURE loss, facilitating the optimization of network parameters.

Our first denoising pipeline, as depicted in Fig. 2, utilizes both b50 and b1000 images as network input, with the b50 images serving as guidance. The low b-value images are integrated into the network input to enhance the denoising process, leveraging their inherently higher SNR and improved delineation of anatomical structures. Preceding network input, a series of preprocessing steps, including phase correction, are applied. We implement a phase correction method proposed by Prah et al.^10^ that can be easily incorporated into any MRI data reconstruction algorithm. First, both the real and imaginary images are spatially filtered, using a uniformly weighted convolution kernel to generate low-frequency phase images. Using the low-frequency phase images, the complex-valued images are rotated by complex rotation. Subsequently, it is assumed that the phase-corrected imaginary image exclusively comprises noise and can therefore be discarded. The remaining phase-corrected real image retains the signal of interest, alongside white noise adhering to the original Gaussian noise distribution, a crucial factor for the applicability of the SURE loss.

After phase correction, we calculate the noise map which is required to compute the SURE loss. This involves computing the standard deviation among the repeated images for each diffusion direction, resulting in one distinct noise map for each direction. Due to the limited number of image repetitions for the b50 data, the calculating a reliable noise map becomes challenging. Therefore, this experiment is specifically focused on denoising high b-value data.

Given the notably low signal-to-noise ratio (SNR) in the individual images and their insufficient signal content, denoising the individual image repetitions would result in blurred outcomes. Therefore, we generate an average image for each diffusion direction using the arithmetic mean. To account for the averaging operation, the noise map is scaled by a factor of $\frac{1}{\sqrt{N}}$, with *N* representing the number of image repetitions within each diffusion direction. Further, the noise map is blurred using a Gaussian filter ($\sigma =10$) to focus on the overall noise distribution rather than local high-frequent changes.

**Fig. 3:**
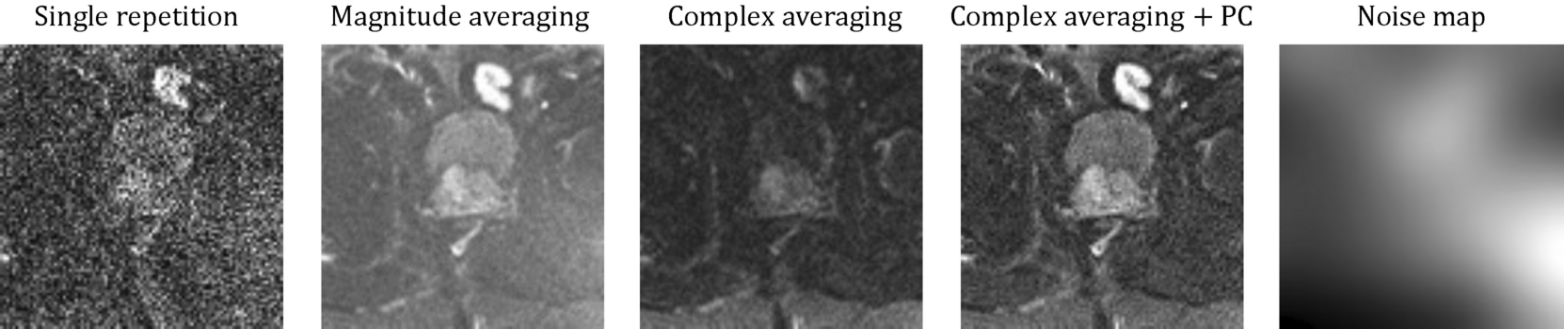
The impact of different processing techniques on the fastMRI b1000 data. The first image shows a single repetition (magnitude image) exhibiting low SNR. Then, we show three different ways of computing the trace image: using the conventional magnitude averaging, complex averaging and complex averaging in combination with a phase correction (PC). For comparison, all trace images are displayed within the same intensity range. The last image shows a noise map, indicating the noise level with pixel accuracy derived directly from the image repetitions.

The impact of our proposed preprocessing techniques is visually demonstrated in Fig. 3. In the case of a single repetition, the high noise level poses a challenge for practical application, diminishing the clinical value of the image. Complex averaging introduces signal loss attributed to phase instabilities, while magnitude averaging accentuates both a raised noise floor and a perceptible fog-like artifact due to the non-zero-centered noise in magnitude images. To mitigate these effects, we propose incorporating complex averaging alongside a phase correction, effectively mitigating unwanted artifacts. Furthermore, we provide a representative noise map for reference that visualizes the inhomogeneous spatial noise distribution.

Following the preprocessing steps, a paired data set consisting of images and their respective noise maps is obtained for each diffusion direction of the b1000 data. As presented by Kaye et al.^5^, the preprocessed low b-value images are input to the denoising network as additional channels to serve as guidance. In this context, a DnCNN architecture, as originally proposed by Zhang et al.^33^, is implemented. By utilizing individual images for each diffusion direction as input instead of computing a trace image, we preserve the Gaussian noise distribution necessary for applying the SURE loss. Subsequently, we optimize the network parameters by minimizing the SURE loss, which is obtained as a single averaged value across all diffusion directions.

#### Using noise maps derived from the scanner output

To address scenarios where an adequate number of repetitions is unavailable for computing the noise map through standard deviation, additional information from the scanner becomes crucial. In our previous study^29^, we presented a method to generate an accurate noise map by propagating a noise prescan, that is routinely acquired as part of the scanner adjustments, through the entire reconstruction pipeline, accounting for factors such as the g-factor map and bias field.

**Fig. 4:**
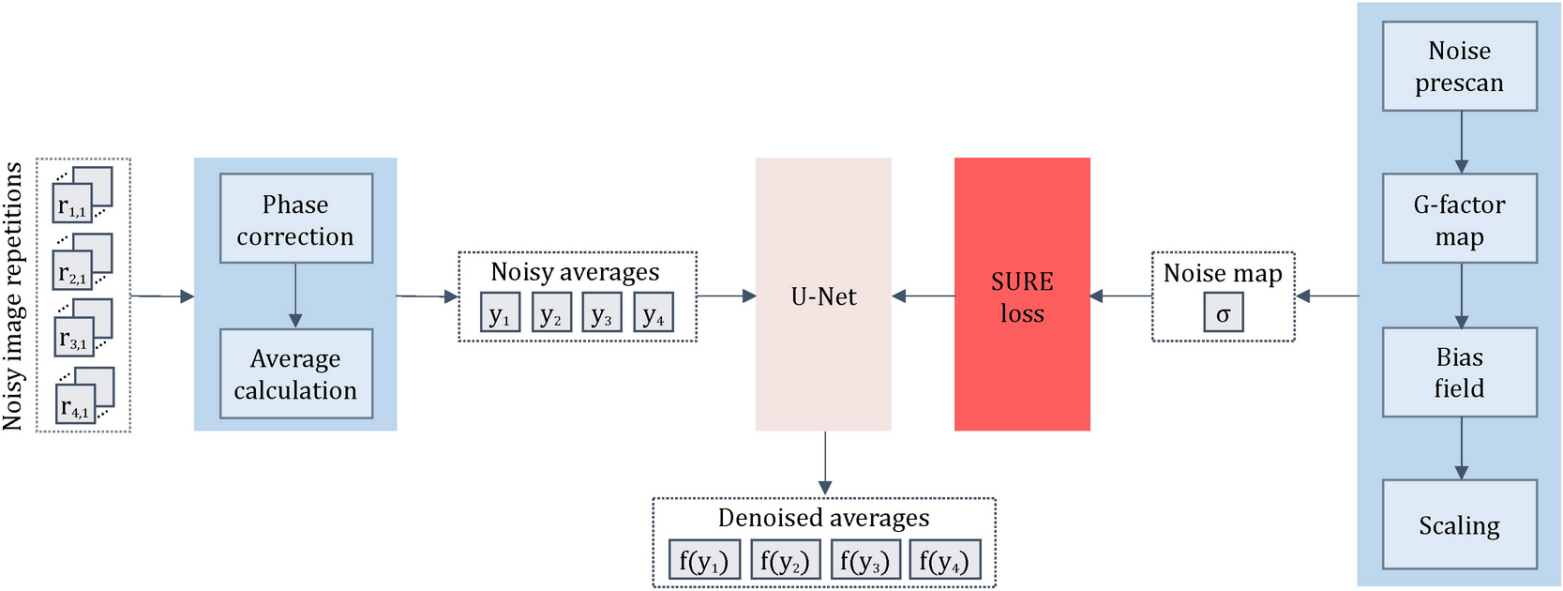
The proposed pipeline using a dedicated noise prescan as additional input. The denoising process initiates with phase correction applied to all image repetitions, followed by generating averaged images for each of the four diffusion directions. These averaged images serve as input for the U-Net denoising network, which is trained through a self-supervised approach utilizing an extended SURE method tailored for spatially variant Gaussian noise. The SURE method incorporates a noise map that represents the pixel-wise noise standard deviation. This map is derived from a dedicated noise adjustment scan conducted during scanner calibration, taking into consideration the g-factor map, bias field, and scaling factors to accommodate phase correction and the effects of averaging.

The denoising pipeline for this strategy is presented in Fig. 4. Due to the necessity of accessing the noise prescan and scanner information, we used the in-house data, specifically the second and third data sets described in Table 1, as input to this pipeline. Similar to the approach presented in the previous section, the denoising pipeline commences with the implementation of phase correction across all image repetitions, followed by the generation of averaged images for each of the four diffusion directions. These averaged images are input to the U-Net architecture^34^, which is trained in a self-supervised manner utilizing our extended SURE method tailored for spatially variant Gaussian noise. The noise map required for this strategy is derived from a dedicated noise adjustment scan conducted during the initial calibration of the scanner, considering the g-factor map, bias field, and scaling factors, to appropriately address the effects of phase correction and image averaging. In this scenario, b50 and b1000 images are denoised separately using a shared network.

**Fig. 5:**
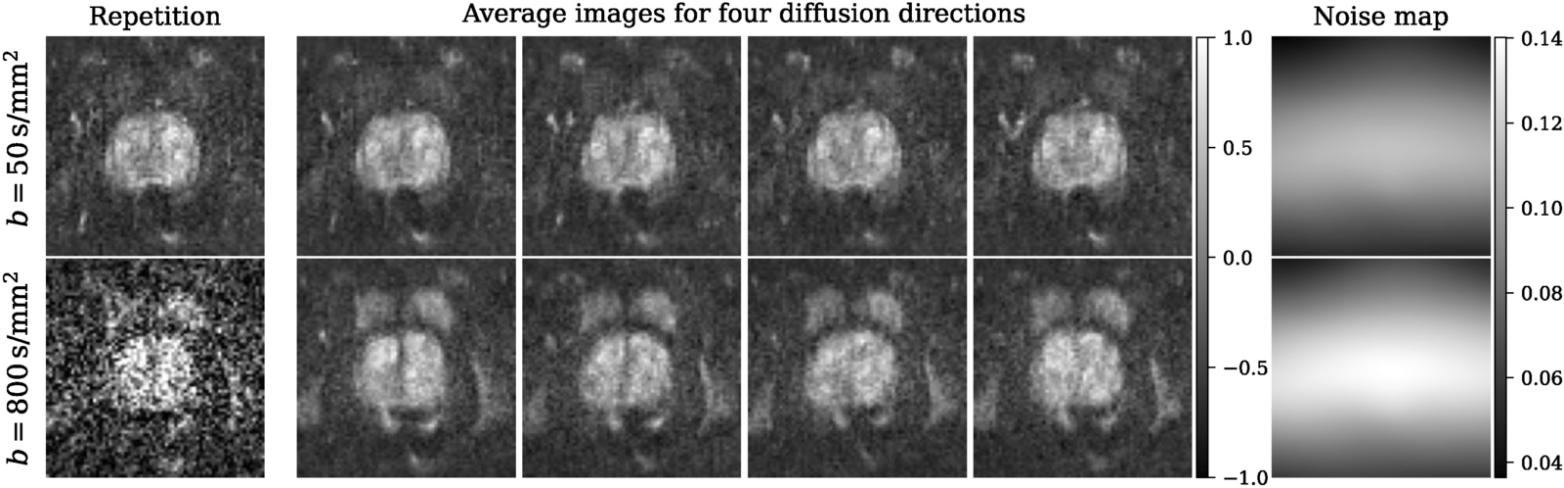
The first column shows a 0.55 T image from a single repetition for both b-values, b50, and b800. Notably, the b800 repetition demonstrates particularly low SNR. In the second through fifth columns, averaged images for the four distinct diffusion directions are displayed. These images are derived from two repetitions for b50 and 15 repetitions for b800 (instead of the routinely acquired four and 22 repetitions, respectively), serving as the input to the network. Furthermore, corresponding noise maps are presented, detailing the standard deviation of noise for each pixel. The noise maps were derived from a dedicated noise prescan.

Representative images illustrating the single repetitions, averaged images for each diffusion direction, and computed noise maps for both b-values are presented in Fig. 5.

### Self-supervised evaluation

Often images computed from a higher repetition count serve as ground truth in cases where no clean data is available. However, these images inherently contain noise, potentially causing inaccuracies when calculating quantitative metrics. To mitigate this, we propose a novel approach that leverages the computed noise maps indicating the noise standard deviation with pixel accuracy. For optimal denoising performance, the residual noise removed by the denoising operator, i.e., the difference between noisy input and denoised output, should entail only pure, spatially variant Gaussian noise. Thus, after being divided by the computed noise map, the corrected residual should be perfectly uniform and follow a Gaussian distribution with a variance of one. To assess this desired state, we compute the Gaussian log likelihood and the variance of the corrected residuals. In the optimal case, 1) the log likelihood should be large and 2) the variance should be as close to one as possible.

## Experiments and results

In this section, the conducted experiments and corresponding results are presented. All CNN models were trained without BatchNorm layers in PyTorch using the Adam optimizer with default parameters, learning rate $5\cdot 10^{-5}$, and minibatch size 64. The DnCNN model comprises a total of 300,675 trainable parameters (1.15 MB), while the U-Net has 17,259,332 trainable parameters (65.86 MB). The training was terminated once the validation loss reached convergence. The training runs were conducted on an internal cluster, consisting of an NVIDIA DGX-1 with 8 Tesla V100 32 GB GPUs and an NVIDIA DGX A100 with 8 A100 40 GB GPUs, with one GPU employed for each training run. The training duration varied, taking up to 6 hours depending on factors such as the model type and dataset. Additionally, the total computational time may vary based on the hardware capabilities. The code for the proposed method is available at https://github.com/laurapfaff201/dwi-denoising to facilitate reproducibility of the research.

### Comparing different denoising methods to enhance the full number of image repetitions

In this experiment, our primary focus lies in enhancing the image quality of a b1000 prostate DWI scan, employing the complete set of repetitions. In this context, we perform a comparative analysis involving four distinct methods, including our proposed denoising pipeline, as depicted in Fig. 2. To ensure a fair comparison, we apply phase correction and complex averaging to all methods, while incorporating the low b-value data as guidance.

Our first comparison method, known as Noise2Noise^22^, entails partitioning the image repetitions into input and reference sets. Subsequently, we compute averaged images for each direction and input these images into the network. A loss is computed by evaluating the network’s output against the averaged images derived from the reference repetitions. Additionally, we explore the MPPCA denoising approach, as originally proposed by Veraart et al.^18^, utilizing the publicly available code repository. Further, we employ a denoising technique inspired by the training strategy presented by Kaye et al.^5^, which we denote as *Half2Full* for the purpose of this work. This method involves processing average images generated from 50% of the repetitions as input, and the MSE loss is determined by comparing the network’s output to the averages computed from the complete set of repetitions. Subsequently, we apply the Half2Full method to the test data, utilizing the complete set of repetitions as input.

**Fig. 6:**
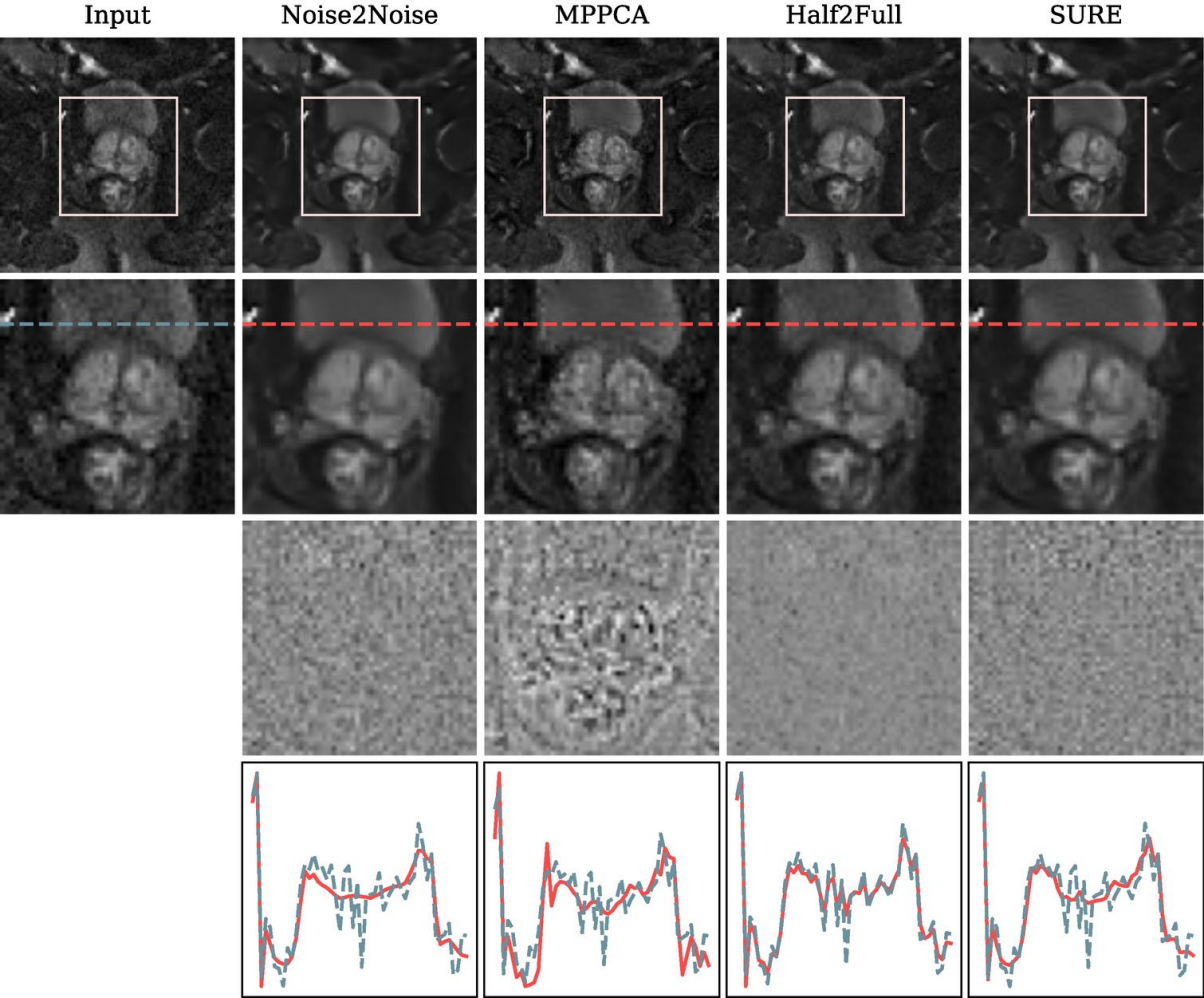
Comparison of different denoising methods using the full number of repetitions included in the fastMRI data set acquired at 3 T. The top row exhibits the b1000 images and the corresponding region of interest (ROI) displayed in the second row. In the third row, the residual noise, i.e., the difference between the noisy and denoised images, is presented. The last row depicts the pixel intensities of the noisy input and the respective denoising result along the lines presented in the second row. Our SURE-based approach excels in effectively removing noise from the image while preserving image sharpness. In the pixel intensity plot, it is clear that the denoised pixel intensities exhibit peaks aligned with those of the noisy pixel intensities, albeit with a noticeably smoother curve.

The visual results of this comparison are depicted in Fig. 6. The outcome achieved using the Noise2Noise technique presents a notably smooth image with finer structures appearing blurred, which is consistent with the substantial noise reduction evident in the residual image. Furthermore, within the pixel intensity plot, it is observable that Noise2Noise excessively smooths the image content, leading to the omission of considerable peaks. The MPPCA method enhances sharpness but concurrently introduces some artifacts, leading to noticeable alterations in specific pixel values as shown in the difference image. Similarly, within the corresponding pixel intensity plot, the outcome based on the MPPCA method displays peaks in the opposite direction, implying a modification of the image content. In contrast, the Half2Full method manages to reduce noise to some degree, although residual noise remains perceptible in the denoised image. This observation is reinforced by the corresponding pixel intensity plot, where the pixel values in the denoised image closely resemble those in the noisy input image version. Our proposed SURE-based method stands out by successfully eliminating the noise from the image without compromising image sharpness. In the plot depicting pixel intensity, it is evident that the peaks of the denoised pixel intensities align with those of the noisy pixel intensities, albeit with a noticeably smoother curve. For Noise2Noise, Half2Full, and SURE, the difference image predominantly contains noise signal, which aligns with the desired outcome.

**Fig. 7:**
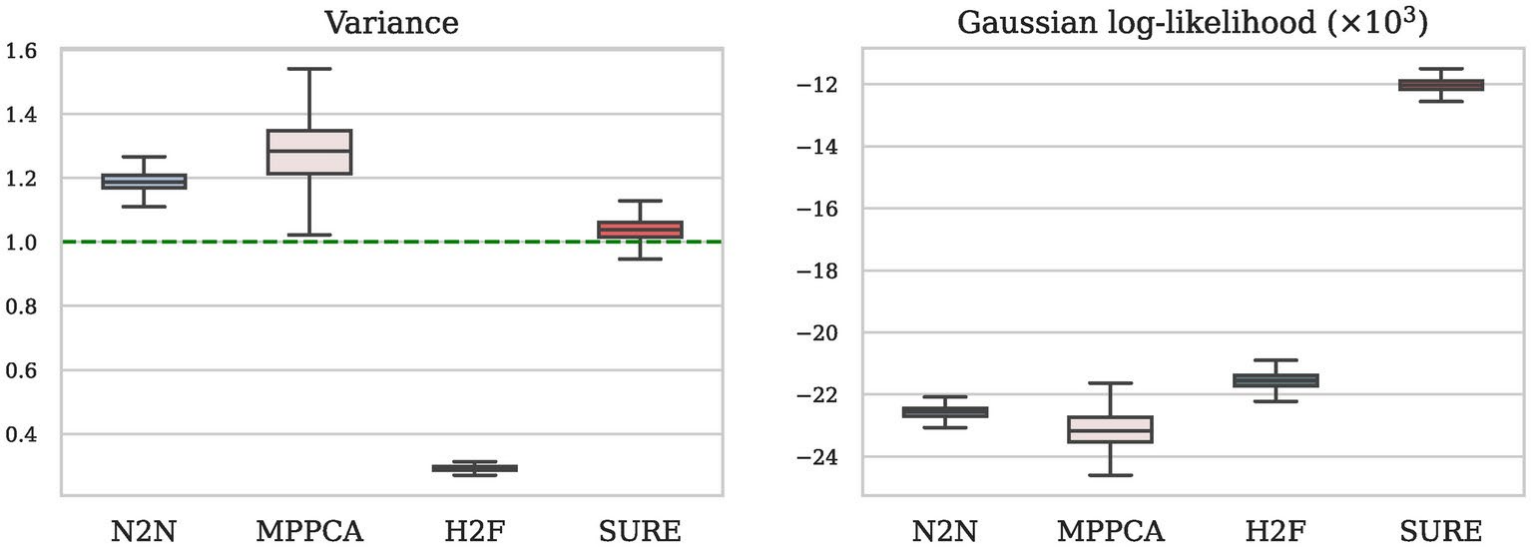
The quantitative evaluation of denoising for fastMRI b1000 images encompasses two aspects: (1) the variance of the corrected residual (optimal value: close to 1.0, represented by the green line), and (2) the Gaussian log-likelihood of the corrected residual. Notably, the SURE method achieves a variance close to 1.0 and the highest Gaussian log-likelihood, signifying that the noise removed by SURE aligns closely with the physical noise model.

The visual findings are further supported by our quantitative evaluation according to section Self-supervised evaluation presented in Fig. 7. The corrected residual generated by the SURE-based model attains a variance closest to one and demonstrates the highest Gaussian log-likelihood. In contrast, both Noise2Noise and MPPCA methods tend to excessively remove image content, while Half2Full fails to eliminate a satisfactory level of image noise.

### Impact of including low b-value images as guidance

In the previous section, we harnessed supplementary information from the lower b-value data (b50) to enhance the denoising of the higher b-value data (b1000). In this experiment, we aim to assess the impact of this supplementary input on the distinct denoising methods. To achieve this, we replicated the experimental conditions outlined in the previous section, without concatenating the b1000 images with the b50 images.

**Fig. 8:**
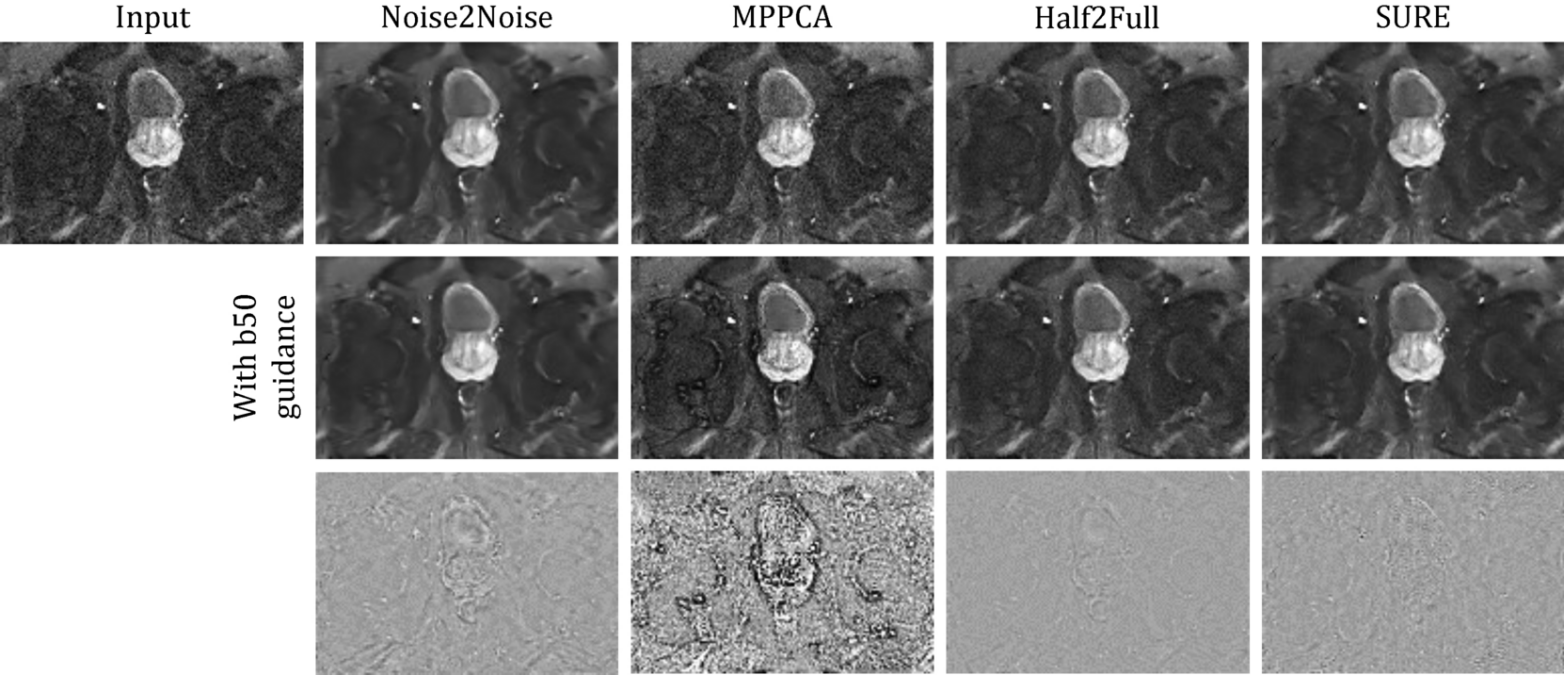
The impact of including the low b-value fastMRI data (3 T). The figure displays denoised b1000 images generated by each method, showing results without the incorporation of b50 data (first row) and with its incorporation (second row). In the third row, the difference between the images when b50 data is included and when it is omitted is presented. While incorporating b50 data considerably impacts the denoising performance of the MPPCA-based method, the difference images indicate a subtle edge enhancement effect for the learning-based methods.

Representative results are depicted in Fig. 8. Upon closer examination, it becomes evident that the MPPCA-based method benefits most from the inclusion of b50 images. Without these additional inputs, the noise reduction achieved in the resulting trace image is minimal. Conversely, in the case of learning-based denoising methods, the inclusion of b50 images appears to play a different yet valuable role. Here, the impact is observed not primarily in noise reduction but in the enhancement of edges and the delineation of tissue boundaries. This results in images that exhibit sharper details and more pronounced structural features.

### Reducing the number of image repetitions

**Fig. 9:**
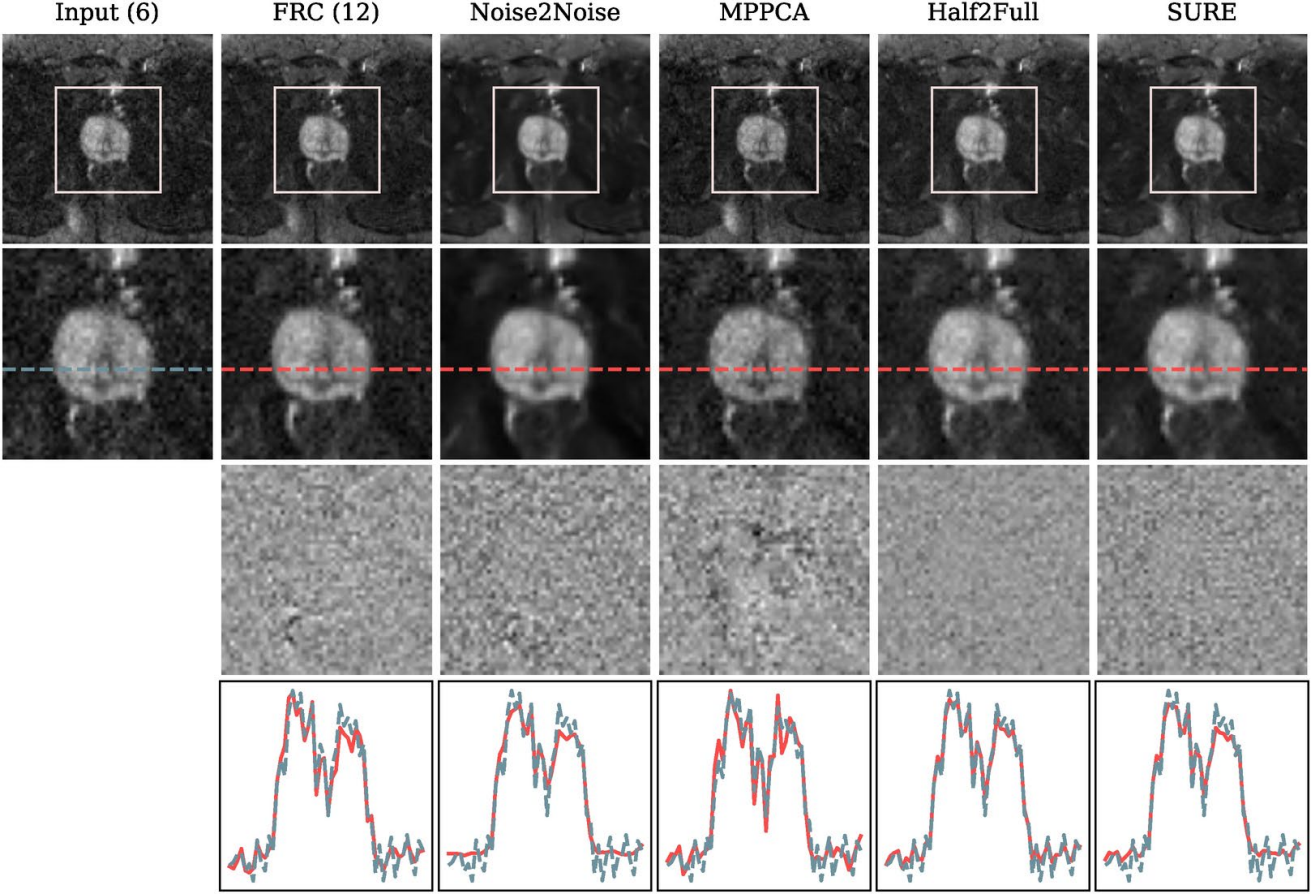
Comparison of different denoising methods using 50% of the available image repetitions included in the fastMRI data set (3 T). The upper row displays both the b1000 images and their corresponding ROI, which is presented in the second row. FRC denotes the trace image using the full repetition count (12) per diffusion direction, while the network input was derived from six repetitions per diffusion direction. The third row shows the residual noise, which displays the difference between the noisy and denoised images. The final row depicts the pixel intensities of both the noisy input and the corresponding denoising results, along the lines presented in the second row.

Given that the number of acquired repetitions has a direct impact on the required scan time, it is a common goal to minimize repetitions while maintaining diagnostic quality. In this experiment, we reduced the number of acquired repetitions by 50%, specifically to two repetitions per direction for b50 and sic repetitions per direction for b1000. We applied the Noise2Noise and Half2Full techniques using the trained models introduced in Section Comparing different denoising methods to enhance the full number of image repetitions, as they already employed averaged images derived from 50% of the repetitions as input by design. Conversely, the SURE-based model was retrained to accommodate the increased noise level.

The resulting denoised images are displayed in Fig. 9. Similar to the previous experiment where the full set of repetitions was used, Noise2Noise produces results with excessive smoothing, leading to the attenuation of brightness in certain image regions. In contrast, the outcome using the MPPCA approach exhibits increased contrast levels and slight alterations in image content. Half2Full and SURE achieve similar results, although the Half2Full outcome exhibits a higher noise level. When comparing the SURE-based result with the reference, which displays the trace image employing the complete set of image repetitions, it becomes evident that no critical image features were removed, and, in fact, the noise level is noticeably diminished.

### Application to low-field and low b-value DWI denoising

To enable the denoising of low b-value data, where the available number of repetitions is typically not sufficient to compute the noise map via standard deviation, we use the pipeline illustrated in Fig. 4. In this experiment, we trained a network on the training data set described in section Data consisting of 1.5 and 3 T scans, and subsequently applied it to the test data set comprising 0.55 T low-field data to demonstrate the robustness of our method. For our test data, we decreased the repetition count to two for b50 and 15 for b800, resulting in a scan time reduction from 7:00 min to 4:47 min.

**Fig. 10:**
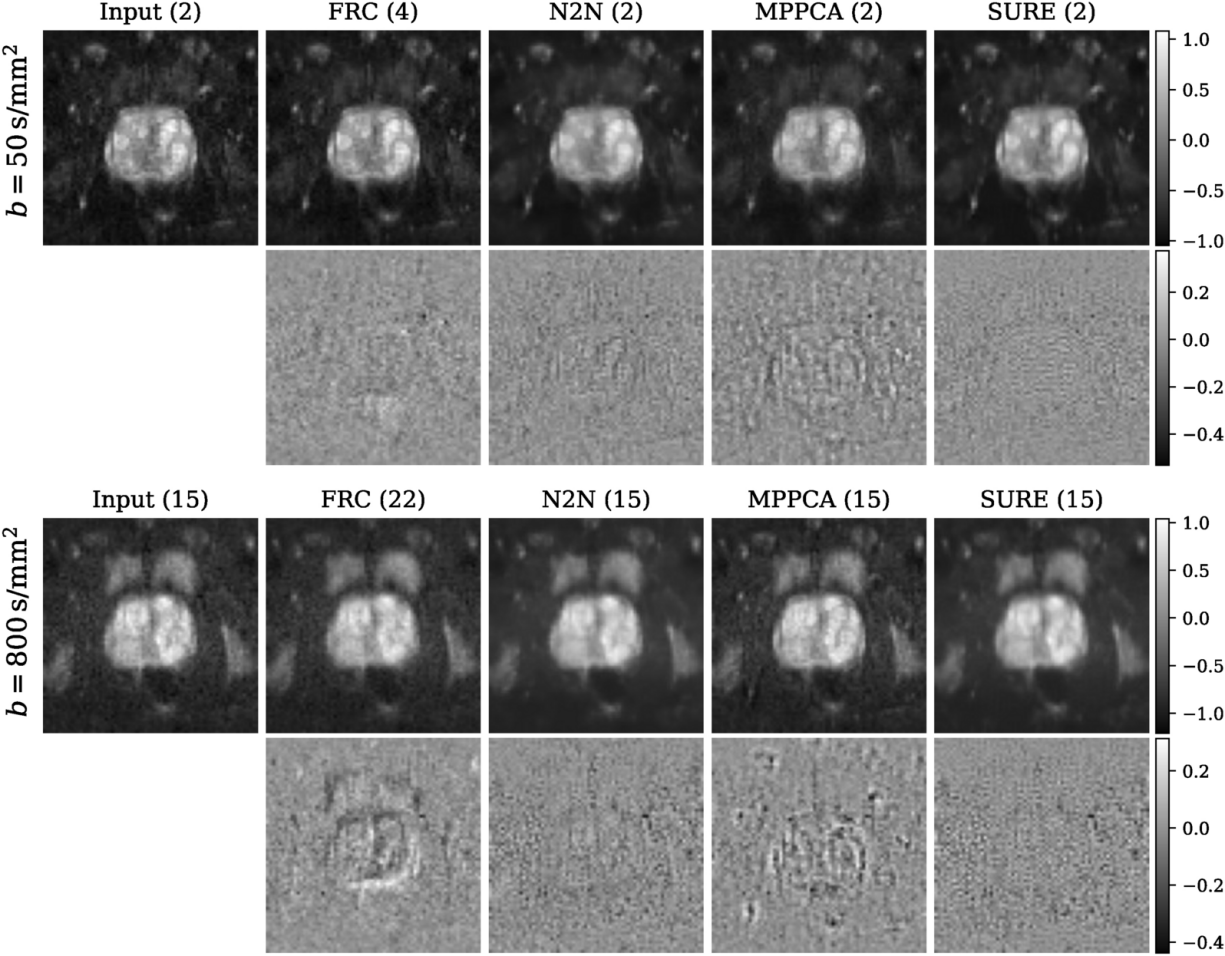
Visual assessment for both b50 and b800 0.55 T images. The first row exhibits the noisy/denoised trace images, with the repetition count per diffusion direction indicated in brackets. The second row displays the difference (i.e., residual) images with respect to the input, all displayed within the same intensity range. The image recovered from the full repetition count (FRC) exhibits considerable alterations of image content, potentially attributed to motion. Conversely, the SURE result showcases efficient noise reduction without any apparent loss of image content.

Figure 10 depicts representative visual results. Increasing the number of repetitions enhances the SNR but introduces content alterations due to patient motion. Despite being trained on high-field data, our SURE-based method effectively reduces noise without compromising image content or blurring high-frequency structures. In contrast, the Noise2Noise residual image, and notably, the MPPCA result, exhibits noticeable structural artifacts.

**Fig. 11:**
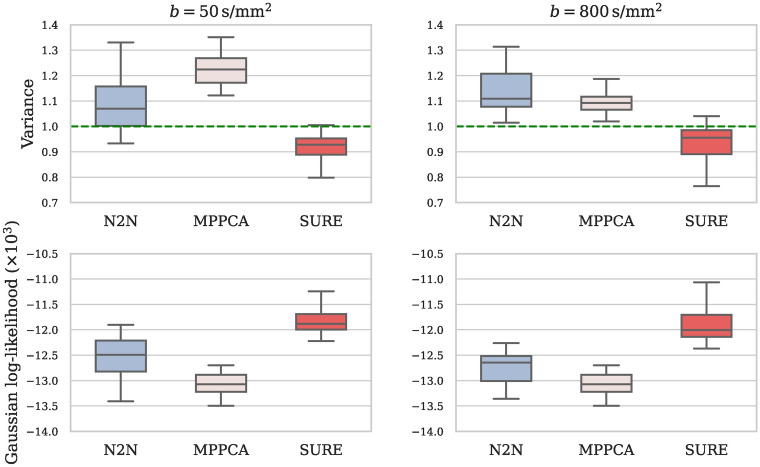
Quantitative assessment for both b-values (b50 and b800) of the 0.55 T in-house data set includes (1) the variance of the corrected residual (ideal value: close to 1.0, denoted by the green line) and (2) the Gaussian log-likelihood of the corrected residual. Remarkably, SURE attains a variance close to 1.0 and the highest Gaussian log-likelihood, indicating that the noise eliminated by SURE closely adheres to the physical noise model.

Quantitative denoising evaluation as described in section Self-supervised evaluation is presented in Fig. 11. Our SURE-based approach produces a corrected residual with a variance close to 1.0 and tends to be more conservative in noise removal. Conversely, the Noise2Noise and MPPCA methods tend to excessively denoise images, potentially compromising image content. Additionally, the Gaussian log-likelihood of the corrected residual is observed to be highest for the SURE-based approach.

### Evaluation of model robustness

**Fig. 12:**
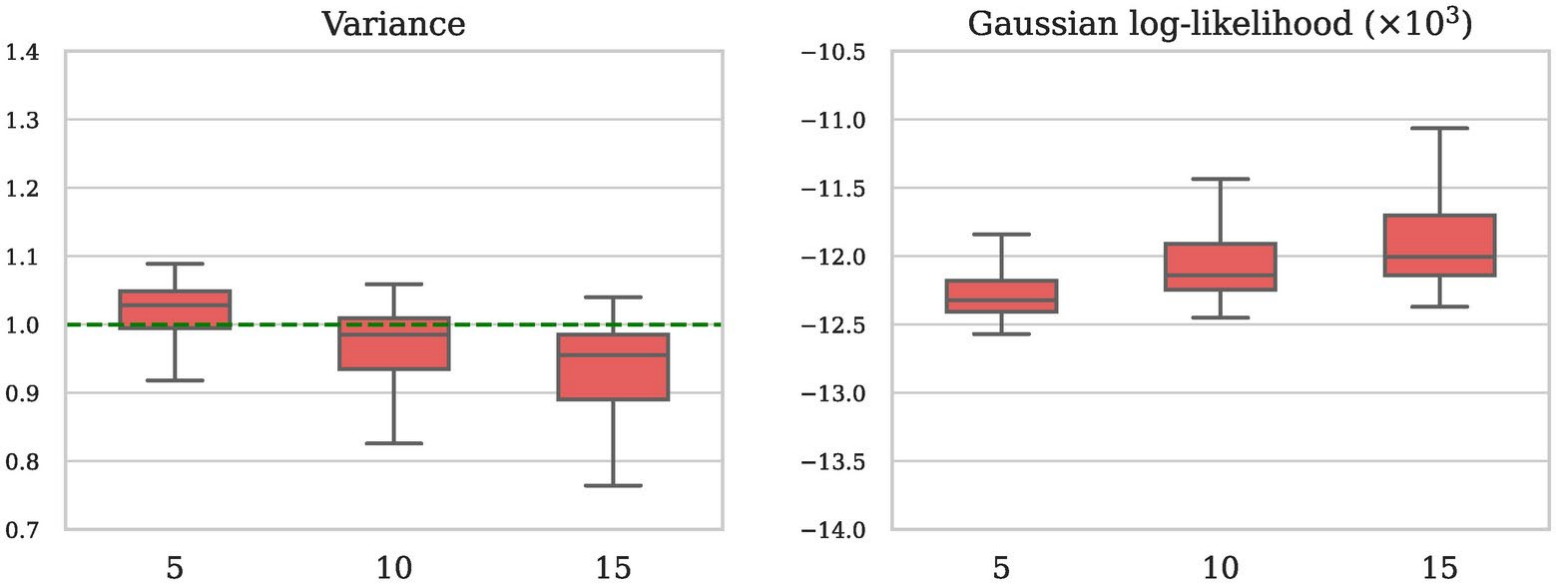
Quantitative assessment of the SURE-based model on the b800 0.55 T in-house data set for a varying number of image repetitions (5, 10, and 15). Left: the variance of the corrected residual (ideal value: close to 1.0, denoted by the green line). Right: the Gaussian log-likelihood of the corrected residual. As the number of image repetitions increases, the variance decreases slightly, while the Gaussian log-likelihood shows a slight increase.

**Fig. 13:**
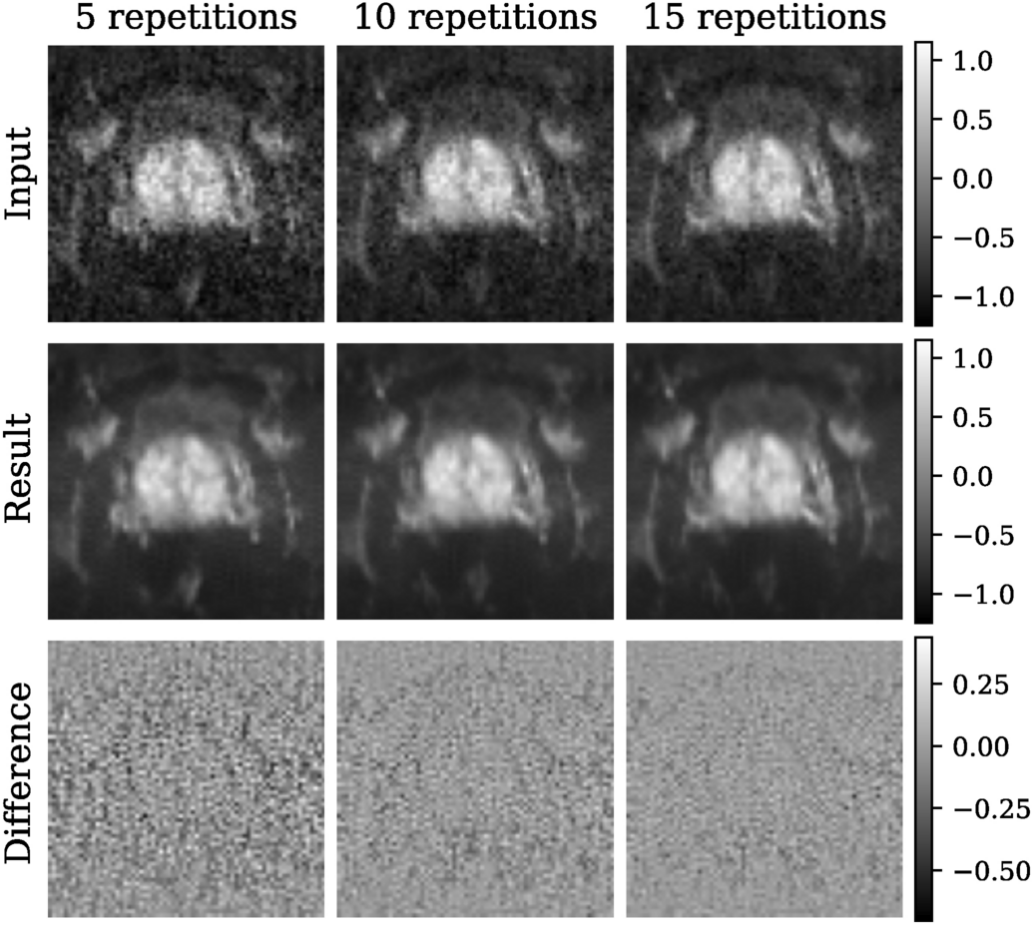
Representative b800 0.55 T image slices including 5, 10, and 15 repetitions per diffusion direction. The model, trained exclusively on higher field data using our proposed SURE-based methodology, successfully delivers effective denoising results across all tested variations.

To provide a more comprehensive understanding of our method’s performance, we evaluated the model trained on higher field data (1.5 and 3 T) using different numbers of image repetitions of the low-field b800 data. This approach allowed us to assess the robustness of the model under varying noise conditions. The quantitative results are presented in Fig. 12, while representative visual results are provided in Fig. 13. Despite being trained on a different data distribution, the model successfully denoises all configurations, demonstrating considerable flexibility with respect to different noise levels, with only very slight changes in quantitative performance.

## Discussion

SURE can be effectively employed for denoising prostate DWI images without the need for noise-free targets, showcasing superior performance compared to state-of-the-art approaches. In our study, we introduced two distinct methods for computing the required noise map. The first method involves calculating the standard deviation across repetitions, offering the benefit of not requiring any additional information—only the images themselves are sufficient for the computation. The second approach employs the propagation of a dedicated noise scan through the entire image reconstruction pipeline. While this method provides an advantage in scenarios with fewer image repetitions, such as low b-value data acquisition, it comes with the drawback of requiring access to specific scanner information.

The effectiveness of the Half2Full principle in denoising DWI images is limited by the number of image repetitions, as it relies on the SNR improvements achievable with the full repetition count. By design, the MPPCA method produces an average image when no second b-value is included. This observation suggests a limited benefit of the MPPCA method in scenarios where a comprehensive set of b-values is not available.

Low b-value images naturally contain a higher signal and, consequently, more distinct edges. This characteristic proves beneficial for guiding the denoising of higher b-value images. However, this enhancement also introduces a potential drawback by altering the image content beyond the scope of denoising based on supplementary data. Such alterations have the potential to influence the diagnosis derived from the resulting images.

An important finding of our study is the potential for a substantial reduction in the number of image repetitions while maintaining clinical value. This has implications for improving patient experience and decreasing scan time without compromising diagnostic accuracy. Additionally, it limits motion artifacts in the averaged images due to misalignment between image repetitions.

We underscored the robustness of our approach by demonstrating its applicability to low-field (0.55 T) scans with a varying number of image repetitions. The model, originally trained on higher field (1.5 and 3 T) data, exhibits consistent and effective denoising capabilities in this lower field-strength setting. This emphasizes the versatility and transferability of our proposed method across different imaging environments.

Evaluating denoising methods presents challenges, particularly in the absence of clean ground-truth data. The subjective nature of readers’ perceptions regarding image noise and sharpness further complicates the assessment. Noise can sometimes enhance the perceived sharpness of images. Consequently, relying on reader studies may not provide an ideal evaluation framework for denoising methods. To address this, our study analyzes the removed noise, providing valuable insights and quantitative measures for a more comprehensive evaluation. By assessing the Gaussianity and variance of the residual image, our evaluation method inherently examines whether the network preserves low-frequency image content, ensuring that essential features are retained throughout the denoising process.

While our proposed denoising method shows promise in improving the quality of DWI images, the assessment of its clinical impact remains an area for future exploration. Despite the initial training time, the inference time for each image is relatively short, making the method suitable for clinical applications where quick results are essential. Additionally, the model size, especially in the case of DnCNN, is manageable, allowing it to be deployed on standard clinical hardware.

## Conclusion

This work employs deep learning-based denoising in a domain where such approaches are underexplored, primarily due to the scarcity of noise-free ground-truth data. We introduced a novel and entirely self-supervised approach for denoising prostate DWI images, particularly emphasizing the denoising of high b-value images with elevated noise levels. The SURE loss is notably well-suited for DWI as it leverages the inherent acquisition of image repetitions, providing a noise map as a byproduct. We further highlighted the versatility of our method by demonstrating how its applicability can be further enhanced through the inclusion of supplementary information from the scanner. Additionally, the utilization of shared information across acquired b-values was investigated, contributing to enhanced denoising performance. Through comprehensive experiments on different data sets, we demonstrated the superiority of our proposed method over state-of-the-art self-supervised learning-based and non-learning-based techniques. We further presented the applicability of our method to accelerate prostate DWI scans by acquiring fewer repetitions or to enhance images with the conventionally acquired repetition count. Furthermore, we introduced a methodology for evaluating denoising performance, even in the absence of noise-free ground-truth data, addressing a critical challenge in the evaluation of self-supervised methods in medical imaging.

## Data Availability

The fastMRI data set is an open-source data set, which contains raw and DICOM data from MRI acquisitions of knees, brains, and prostate DWI, and can be obtained from the NYU fastMRI initiative database^30–32^. The remaining data sets were acquired by Siemens Healthineers and are not publicly available. The data are however available from the authors upon reasonable request and with permission of Siemens Healthineers. The point of contact for inquiries regarding the dataset is Laura Pfaff, and can be reached via email at laura.pfaff@fau.de.
